# List of Diseases at the Bath City Dispensary, from May 1, to June 1, 1802

**Published:** 1802-07

**Authors:** 


*List of Diseases at the Bath City Dispensary, from May* 1, *to June* 1, 1802.

| | | | |
|---|---|---|---|
| Angina | 3 | Menorrhagia | 2 |
| Afthma | 1 | Ophthalmia | 2 |
| Abfcefs | 1 | Pleuritis | 3 |
| Anafarca | 2 | Phthifical | 6 |
| Afcites | 1 | Pulmonary Complaints | 2 |
| Afthenia | 1 | Pertuffis | 1 |
| Bilious Complaints | 3 | Paralytic | 1 |
| Catarrh | 2 | Pleurodyne | 3 |
| Contufion | 3 | Prurigo | 1 |
| Chlorofis | 3 | Pforiafis Gyrata | 1 |
| Diarrhœa | 3 | Rheumatifm | 14 |
| Eryfipelas | 1 | Rubeola | 7 |
| Eruptiones | 4 | Synochus Biliofa | 13 |
| Fever | 5 | ——— Pleuritica | 2 |
| ——— Intermittent | 2 | ——— Dyfenterica | 1 |
| Fiftula in Perinæo | 1 | Syphilitic | 2 |
| Gaftrodynia | 4 | Scrophula | 2 |
| Hernia | 2 | Scirrhus | 2 |
| ——— Humoralis | 1 | Tinea | 3 |
| Hæmoptyfis | 1 | Tuffis cum Dyfpnœa | 1 |
| Herpes | 3 | Ulcerated Legs | 3 |
| Ifcuria | 1 | Vertigo | 1 |
| Icterus | 1 | | |
| Infantile Complaints | 3 | Total | 120 |
| Lepra | 4 | | |

